# The many faces of cGAS: how cGAS activation is controlled in the cytosol, the nucleus, and during mitosis

**DOI:** 10.1038/s41392-021-00684-3

**Published:** 2021-07-09

**Authors:** Anna-Maria Herzner, Martin Schlee, Eva Bartok

**Affiliations:** 1 Department of Cancer Immunology and Immune Modulation, Boehringer Ingelheim Pharma GmbH & Co. KG, Biberach an der Riß, Germany; 2 Institute of Clinical Chemistry and Clinical Pharmacology, University Hospital, University of Bonn, Bonn, Germany; 3 Unit of Experimental Immunology, Department of Biomedical Sciences, Institute of Tropical Medicine, Antwerp, Belgium

**Keywords:** Innate immunity, Inflammation

**Fig. 1:**
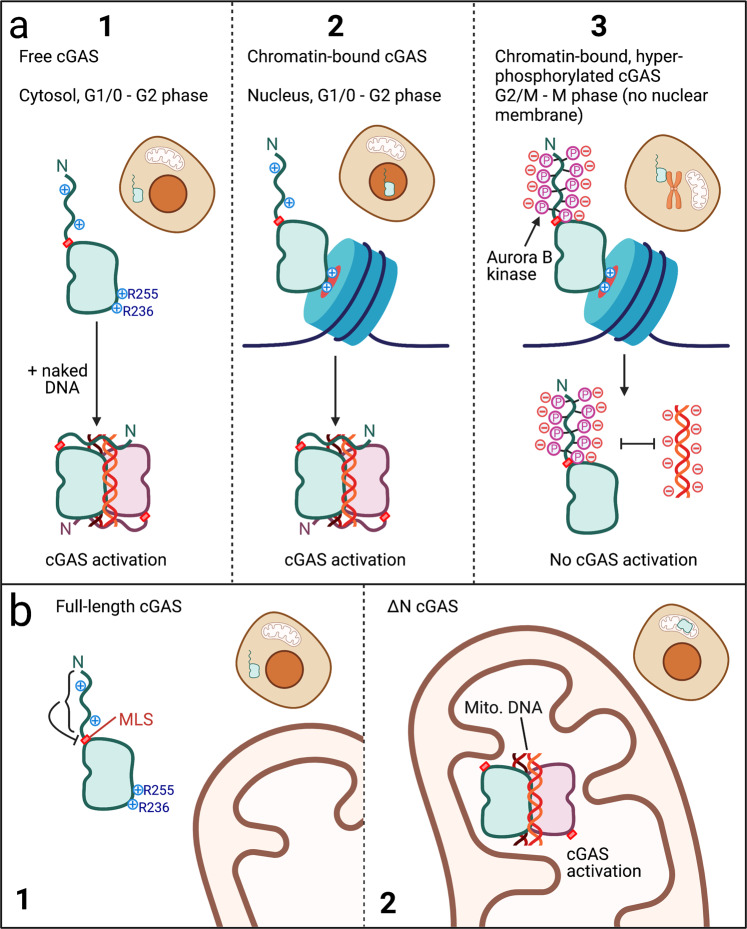
The many faces of cGAS. **a** (1) cGAS was initially discovered as a cytosolic DNA receptor that can be activated by dsDNA >40 bp in a sequence-independent manner. Li et al. demonstrate that cytosolic cGAS is neither chromatin-bound nor hyperphosphorylated at its N-terminus. (2) Subsequent studies demonstrated that cGAS was present in the nucleus and tethered to chromatin to prevent activation with genomic DNA. Li et al. corroborate the R236/R255-mediated binding demonstrated in Volkman et al. and provide evidence that this form of cGAS also has a non-phosphorylated N-terminus and can be activated by exogenous/naked (non-chromatin bound) DNA. (3) During mitosis, the barrier between nucleus and cytosol dissolves. Li et al. demonstrate that this form of cGAS is both tethered to chromatin and phosphorylated at the N-terminus by kinases, including Aurora kinase B. Neither chromatin nor naked DNA can activate this form of cGAS. **b** (1) The N-terminus of full-length cGAS or N-terminal tags, such as 3xFLAG, mask a cryptic mitochondrial localization sequence (MLS) preventing its mitochondrial localization. (2) Removing the N-terminus results in mitochondrial localization and constitutive activation of cGAS by mitochondrial DNA

In their recently published study in Science, Li et al.^1^ unravel how hyperphosphorylation and chromatin-tethering of the double-stranded (dsDNA) sensor cGAMP synthase (cGAS) prevent self DNA recognition during mitosis (Fig. 1), providing important insight into how this innate immune sensor balances its essential function for pathogen defense against potential autoinflammation.

dsDNA over 40 bp and guanosine-bearing, Y-form DNA activate cGAS, a nucleotidyltransferase that generates its cognate dinucleotide, 2′–3′ cGAMP, which then activates Stimulator of Interferon Genes (STING), downstream IRF3 and NF-KB signaling, and the release of proinflammatory mediators and type-I interferon (IFN).^2^ This pathway is a major workhorse of the antiviral response, responsible for sensing a number of DNA viruses, bacterial and protozoan pathogens, DNA from damaged or malignantly transformed cells, and, indirectly, any pathogens inducing mitochondrial damage and DNA release, including RNA viruses. cGAS or STING-deficient mice succumb to poxviruses and HSV-1, demonstrating the essential role of this pathway in vertebrate immune defense. Nonetheless, key aspects of cGAS regulation have remained unclear, in particular how cGAS is shielded from constitutive activation by self DNA in the nucleus and during mitosis.

With their recent publication, Li et al. contribute to our understanding of the nuclear and mitotic regulation of cGAS signaling.^1^ In line with previous reports, their study confirms that a large proportion of cellular cGAS is nuclear and that its tethering to chromatin limits its nuclear activation by genomic DNA.^3,4^ Moreover, the authors describe a novel state of mitotic cGAS regulation (Fig. 1a), in which, in addition to tethering, cGAS is completely inactivated by phosphorylation of its N-terminal domain.

The study begins with an in vitro examination of cGAS during cell cycle phases. Here, Li et al. observed that, unlike cGAS extracted from the nucleus and cytoplasm of cells in the G1 and S phase, mitotic cGAS (G2/M and M phase) was incapable of producing cGAMP upon herring testes (HT-)DNA stimulation, thus providing evidence for a distinct “mitotic” cGAS state. As phosphorylation is a major determinant of cell cycle progression, they then investigated the phosphorylation status of cGAS in cells blocked at the G2/M border by CDK1 inhibition. G2/M blockage held cGAS in a phosphorylated state which resolved with blockage release and cell cycle progression. Mass spectrometry revealed 9 phosphorylation sites appearing during G2/M within the cGAS N-terminus. Moreover, Li et al. could also identify one kinase involved in cGAS phosphorylation, Aurora B kinase, a known mediator of cell cycle progression.

The role of the highly basic, unstructured N-terminus (1–160 AA) of cGAS remains controversial in the literature. Multiple reports have demonstrated the interaction of the unstructured, positively charged N-terminus with chromatin DNA.^2–4^ It has also been described as a cytosolic retention signal,^3^ and the Chen group has reported that the N-terminus enhances phase condensation and activation of cytosolic cGAS.^5^ Clearly, hyperphosphorylation would profoundly change the charge of this domain, potentially disrupting ionic interactions between cGAS and DNA. In their study, Li et al. demonstrate that the substitution of all 20 potential phosphorylation sites in the N-terminus with phosphomimetic aspartate or glutamate (20DE) could abrogate cGAS activity and phase condensation, suggesting that hyperphosphorylation was indeed responsible for mitotic inhibition. In contrast, substitution with non-phosphorylatable alanine (20A) did not disturb either process, although 20 A still remained inactive during mitosis. However, using a split-GFP reporter, Li et al. could demonstrate that both the wildtype and 20 A mutant failed to oligomerize during mitosis, suggesting that DNA tethering may act as a further mechanism restricting mitotic cGAS activity.

To further investigate this, they utilized two different cGAS mutations, R236E and R255E, previously reported by Volkman et al.^4^ to abrogate tethering to nuclear DNA. Although single mutations of R236E or R255E lead to activation of cGAS in cell lysates, the purified cGAS mutants were inactive without HT-DNA addition, corroborating the report of Volkman et al. that these mutants can be activated by nuclear chromatin. Nevertheless, R236E and R255E were not sufficient for activity during mitosis, instead of requiring a combination with 20 A to restore cGAS activity. Li et al. thus demonstrate that chromatin-tethering and N-terminal hyperphosphorylation act as a dual mechanism to inhibit cGAS during cell division (Fig. 1a).

To better understand its function, the N-terminus was deleted from wildtype (cGAS-ΔN) and the R236E and R255E mutants (cGAS-ΔN^R236E^, cGAS-ΔN^R255E^). cGAS-ΔN^R236E^ and cGAS-ΔN^R255E^ were no longer constitutively active, indicating that this domain is necessary for chromatin DNA sensing. However, after completely removing any tag, Li et al. made the unexpected observation that cGAS-ΔN relocalized to the mitochondria, which could be recapitulated in an AA161-175-GFP fusion protein, indicating that AA161-175 contains an otherwise cryptic mitochondrial localization signal (MLS). In STING-competent cells, tag removal also induced IFN signaling. Although seemingly in conflict with previous studies on the activity of cGAS-ΔN, it should be noted that all of these reports, including Du et al., utilized either cell-free systems or an N-terminal tag (e.g., 3xFLAG), potentially masking the MLS. Although this observation may account for the novel discovery of the MLS in this study, it also underscores the inherent difficulty of functionally investigating the N-terminus and may call into question many of the experiments performed with N-terminally tagged cGAS-ΔN to date.

Clearly, the observations of Li et al. have broad implications for our understanding of cGAS regulation and function. Although a coincidental finding, the discovery of an MLS in cGAS may represent an ingenious mechanism to counter pathogen proteases that could conceivably cleave off the exposed N-terminus. Moreover, Li et al. have demonstrated a novel, direct link between cell cycle control and cGAS activity. Viruses and malignantly transformed cells are known to boost cellular proliferation and thus may also benefit from mitotic cGAS inhibition. Moreover, mitotic cGAS inhibition may also contribute to the anti-inflammatory effects of microtubule inhibitors, such as colchicine, which arrest cells in metaphase. Conversely, cellular senescence can be viewed as a vicious circle, resulting from self-reinforcing increased cGAS signaling and decreased cell division. Altogether, cGAS phosphorylation during mitosis provides a new regulatory layer for an otherwise potentially autoinflammatory pathway. Undoubtedly, future studies will focus on how the de/phosphorylation of cGAS contributes to sterile inflammation and pathogen defense, with an eye to developing new targets and therapies in health and disease.
